# Differences in frontotemporal dysfunction during social and non-social cognition tasks between patients with autism spectrum disorder and schizophrenia

**DOI:** 10.1038/s41598-018-21379-w

**Published:** 2018-02-14

**Authors:** Keiko Hirata, Kazuteru Egashira, Kenichiro Harada, Mami Nakashima, Masako Hirotsu, Shinji Isomura, Toshio Watanuki, Toshio Matsubara, Yoichi Kaku, Hiroshi Kaneyuki, Yoshifumi Watanabe, Koji Matsuo

**Affiliations:** 1 Division of Neuropsychiatry, Department of Neuroscience, Yamaguchi University School of Medicine, Ube, Yamaguchi Japan; 2 Katakura Hospital, Ube, Yamaguchi Japan; 3 Egashira Clinic, Kitakyusyu, Fukuoka Japan; 4 Nagatoichinomiya Hospital, Shimonoseki, Yamaguchi Japan; 5 Yamaguchi Prefectural Mental Health Medical Center, Ube, Yamaguchi Japan; 6 Health Service Center, Yamaguchi University Organization for University Education, Yamaguchi, Yamaguchi Japan

## Abstract

Although literature evidence suggests deficits in social and non-social cognition in patients with autistic spectrum disorder (ASD) and schizophrenia (SCZ), the difference in neural correlates of the impairments between the two disorders has not been elucidated. We examined brain function in response to a non-social cognition and a social cognition task using functional near-infrared spectroscopy (fNIRS) in 13 patients with ASD, 15 patients with SCZ, and 18 healthy subjects. We assessed the brain function of participants using a verbal fluency task and an emotional facial recognition task. The patients with ASD showed significantly reduced brain activation in the left frontotemporal area during both tasks compared to healthy subjects. The patients with ASD with larger score in ‘attention to detail’ in the autism spectrum quotient showed lower activation of the left frontotemporal area during the two tasks. The patients with SCZ showed significantly reduced activation, compared to healthy subjects, and greater activation, compared to patients with ASD, in the area during the verbal fluency task. The patients with SCZ with more severe symptoms had lower brain activation during the task in this area. Our results suggest that two distinct areas are involved in the distinctive brain pathophysiology relevant to cognitive processing in patients with ASD and SCZ.

## Introduction

Autism spectrum disorder (ASD) is characterised by persistent deficits in social communication and social interaction across multiple contexts, including deficits in social reciprocity, nonverbal communicative behaviours used for social interaction, and skills in developing, maintaining, and understanding relationships^1^. Patients with ASD also have restricted, repetitive patterns of behaviour, interests, or activities. The differential diagnosis of ASD is listed among other neurodevelopmental disorders and schizophrenia (SCZ) in the Diagnostic and Statistical Manual of Mental Disorders-fifth edition (DSM-5)^1^ because the social deficits present in ASD resemble the social impairment, atypical interests, and beliefs occurring during the prodromal state in schizophrenia (SCZ). In turn, patients with SCZ often show social dysfunction due to negative symptoms (diminished emotional expression or avolition), which is more common in the chronic stage of the disease^1^. The differential diagnosis of SCZ is listed among other psychotic disorders, ASD, major depressive disorder, and anxiety disorders^1^. Patients with ASD must also have symptoms that meet the criteria for schizophrenia in order to be diagnosed with schizophrenia as a comorbid condition based on DSM-5. Although ASD and SCZ should be clinically discriminated and cause socially and economically poor functional outcome for individuals and significant negative impact on society^2–4^, the distinctive neural mechanisms underpinning the two disorders remain unclear.

Accumulated evidence shows that deficits in social and non-social impairment are shared and neurocognitive dysfunction differs between ASD and SCZ^5–12^. Patients with ASD, for instance, showed a lower score in facial identification and face emotional recognition tasks compared to those with SCZ or healthy subjects^8^. Another study revealed that poor performance in batteries of social and non-social neurocognitive tests was a shared feature in patients with ASD and SCZ^10^. However, to our knowledge, there are few neuroimaging studies evaluating and comparing neural correlates for both social and non-social cognition in the two disorders^13–15^. A meta-analysis of fMRI studies attempted to indirectly infer differences in neural substrates of social cognition between ASD and SCZ based on findings in patients with ASD vs. healthy subjects, and those with SCZ vs. healthy subjects^13^. During facial emotional recognition tasks, patients with ASD, compared to those with SCZ, showed increased brain activation in temporal regions near the superior temporal sulcus and in the anterior and posterior cingulate cortex, and decreased activation in the ventrolateral prefrontal cortex, parahippocampal gyrus, and regions within the temporoparietal junction, inferior occipital gyrus, and the cerebellum. However, to date, there has been no comparative functional neuroimaging study of social and non-social cognition (e.g., executive function) in patients with ASD and those with SCZ.

Functional near-infrared spectroscopy (fNIRS) measures real-time hemodynamic alteration over the surface of the brain with infrared spectrum light. The fNIRS method is noninvasive, relatively insensitive to motion artefacts, and can measure brain function with participants seated in a natural position, with little stress of body restriction during the examination, unlike fMRI which may be stressful to participants. These advantages make fNIRS suitable to assess brain function in subjects with psychiatric disorders such as ASD^16–18^ and schizophrenia^19–23^. Prior fNIRS studies of ASD showed that patients with ASD had blunted activation of the frontopolar prefrontal area during executive tasks (verbal fluency tasks) compared to healthy subjects^16–18^. Similarly, several fNIRS studies of SCZ have consistently shown that patients with SCZ have poor activation of the frontotemporal area during executive tasks^19–21^ and emotional social cognitive tasks^22,23^, compared to healthy subjects. However, a comparative study of patients with ASD and SCZ evaluating social cognition and non-social cognition (e.g., executive function) using fNIRS has not been published. We attempted to advance the evidence regarding frontotemporal dysfunction during facial emotional cognitive tasks in patients with SCZ in our previous studies^22,23^.

Here, we aimed at examining the differences in brain function between patients with ASD and those with SCZ, in response to social and non-social cognition, using fNIRS. Based on literature evidence from fMRI and fNIRS studies in ASD and SCZ, we hypothesised that patients with the two disorders would show lower brain activity in the frontal and the temporal areas during the cognition-based tasks, compared to healthy subjects. We also sought to identify the functional abnormalities which are distinct between the two disorders.

## Results

### Behavioural performance

There was no significant difference in patient clinicopathologic characteristics (age, sex, or premorbid Intelligence Quotient [IQ]) across patients with ASD, patients with SCZ, and healthy subjects (Table 1).

**Table 1:** Demographic and clinical background profiles of study participants.

	ASD (n = 13)	SCZ (n = 15)	Healthy (n = 18)	*p*
Age, years	30 [23.3–38.5]	36 [29–47]	34.5 [28–38.5]	0.18^a^
Sex(M/F)	12/1	12/3	13/5	0.38^b^
JART IQ	106.0 [99.5–115.9]	100.0 [91.3–109.6]	102.9 [94.9–107.9]	0.32^a^
**AQ**				
Social skills	8 [5–9]	—	—	—
Attention switching	8 [5–9]	—	—	—
Attention to detail	5 [3–7]	—	—	—
Communication	6 [4–8]	—	—	—
Imagination	5 [4–6]	—	—	
Total	31 [25–34.5]	—	—	—
**PANSS**				
Positive factor	—	9 [7–10]	—	—
Negative factor	—	14 [12–21]	—	—
General psychopathology	—	21 [19–24]	—	—
Total	—	45 [41–53]	—	—
Chlorpromazine eq.(mg)	0 [0–75]	800 [489.4–1300]	—	<0.01^c^
Imipramine eq.(mg)	38 [6.3–12.5]	0 [0–0]	—	<0.01^c^
Duration of illness(years)	—	11 [8–23]		—

^a^Kruskal-Wallis Test; ^b^Pearson Chi-Square test; ^c^Mann-Whitney *U* Test.

ASD, Autistic spectrum disorder; SCZ, Schizophrenia; M/F, male/female; JART, Japanese version of the National Adult Reading Test; AQ, Autism Spectrum Quotient; PANSS, Positive and Negative Syndrome Scale; Eq., equivalent. The values represent median [inter-quartile range].

For the verbal fluency task as a non-social cognition, the results revealed a significant difference and large effect size in the number of words generated across the three diagnoses (*chi-squared* = 12.7, *df* = 2, *p* < 0.01, *η*^2^ = 0.15) (Table 2). The post-hoc analysis showed a significant difference and large effect size between patients with ASD and healthy subjects (*chi-squared* = 4.43, *df* = 1, *p* = 0.04, *η*^2^ = 0.14), between patients with SCZ and healthy subjects (*chi-squared* = 9.94, *df* = 1, *p* < 0.01, *η*^2^ = 0.31), and between patients with ASD and those with SCZ (*chi-squared* = 4.82, *df* = 1, *p* = 0.03, *η*^2^ = 0.17).

**Table 2:** Results of behavioural performance in the tasks.

		ASD	SCZ	Healthy
**Verbal fluency task**				
	Generated words^a^	16 [10.5–19]^b^	10 [8–14]	11.5 [8.8–16]
**Emotional face recognition task**				
Identification	Accuracy (%)	100 [100–100]	100 [100–100]	100 [100–100]
	MRT (ms)^a^	1483.1 [1431.4–1614.8]^c^	1079.2 [900.8–1593.4]^d^	852.1 [779.4–1006.6]
Emotion	Accuracy (%)	100 [91.7–100]	100 [91.7–100]	100 [100–100]
	MRT (ms)^a^	2001.7 [1784.8–2231.7]^c^	1946.5 [1472.5–2270.3]^d^	1251.5 [1076.6–1426.7]

^a^*p* < 0.01 by Kruskal-Wallis Test. ^b^*p* < 0.05, ASD vs. SCZ; ^c^*p* < 0.01, ASD vs. Healthy; ^d^*p* < 0.01, SCZ vs. Healthy. ASD, Autistic spectrum disorder; SCZ, Schizophrenia; MRT, mean reaction time. The values represent median [inter-quartile range].

For the emotional facial recognition task as a social cognition, the accuracy in the ‘identification’ and ‘emotion’ tasks was not significantly different across the three groups (Table 2). However, the mean reaction time (MRT) showed a significant difference and large effect size across the three groups (‘identification’ and ‘emotion’ tasks: *chi-squared* = 22.2, *df* = 2, *p* < 0.01, *η*^2^ = 0.49; *chi-squared* = 24.8, *df* = 2, *p* < 0.01, *η*^2^ = 0.55, respectively). In the post-hoc analysis, the MRTs of the ‘identification’ and ‘emotion’ tasks showed a significant difference and large effect size between patients with ASD and healthy subjects (‘identification’, *chi-squared* = 20.8, *df* = 1, *p* < 0.01, *η*^2^ = 0.69; ‘emotion’, *chi-squared* = 4.43, *df* = 1, *p* = 0.04, *η*^2^ = 0.14, respectively) and between patients with SCZ and healthy subjects (*chi-squared* = 9.94, *df* = 1, *p* < 0.01, *η*^2^ = 0.31; *chi-squared* = 14.1, *df* = 1, *p* < 0.01, *η*^2^ = 0.44), but not a significant difference between patients with ASD and those with SCZ (*chi-squared* = 2.67, *df* = 1, *p* = 0.10, *η*^2^ = 0.10; *chi-squared* = 0.58, *df* = 1, *p* = 0.45, *η*^2^ = 0.02).

### Cognitive behaviour and clinical association

We also examined the association of the behavioural results and clinical variables with the Spearman’s rho test. Clinical variables consisted of premorbid IQ, the imipramine-equivalents, chlorpromazine-equivalents, and total and five subscales of the autism spectrum quotient (AQ) for ASD and premorbid IQ, the duration of illness, chlorpromazine-equivalents, and total and three subscales of the Positive and Negative Syndrome Scale (PANSS) for SCZ. We converted the medication load prescribed to the patients at the time of study participation into chlorpromazine-equivalents for antipsychotics and imipramine-equivalents for antidepressants^24^. There was no significant correlation between task performance in the verbal fluency task and the clinical variables or medication load in patients with ASD or SCZ. For the emotional face recognition task, there was no significant correlation between MRT and any clinical variable in patients with ASD. In patients with SCZ, the MRT in the ‘emotion’ task correlated significantly and positively with the score pertaining to negative symptoms on the PANSS (*rho* = 0.59, *p* = 0.02), and inversely correlated with the premorbid IQ score (*rho* = −0.56, *p* = 0.029). However, these results did not reach significance after Bonferroni correction. No significant correlation was seen between MRT and medication load or the other clinical variables in patients with ASD or SCZ.

### fNIRS

**Figure 1:**
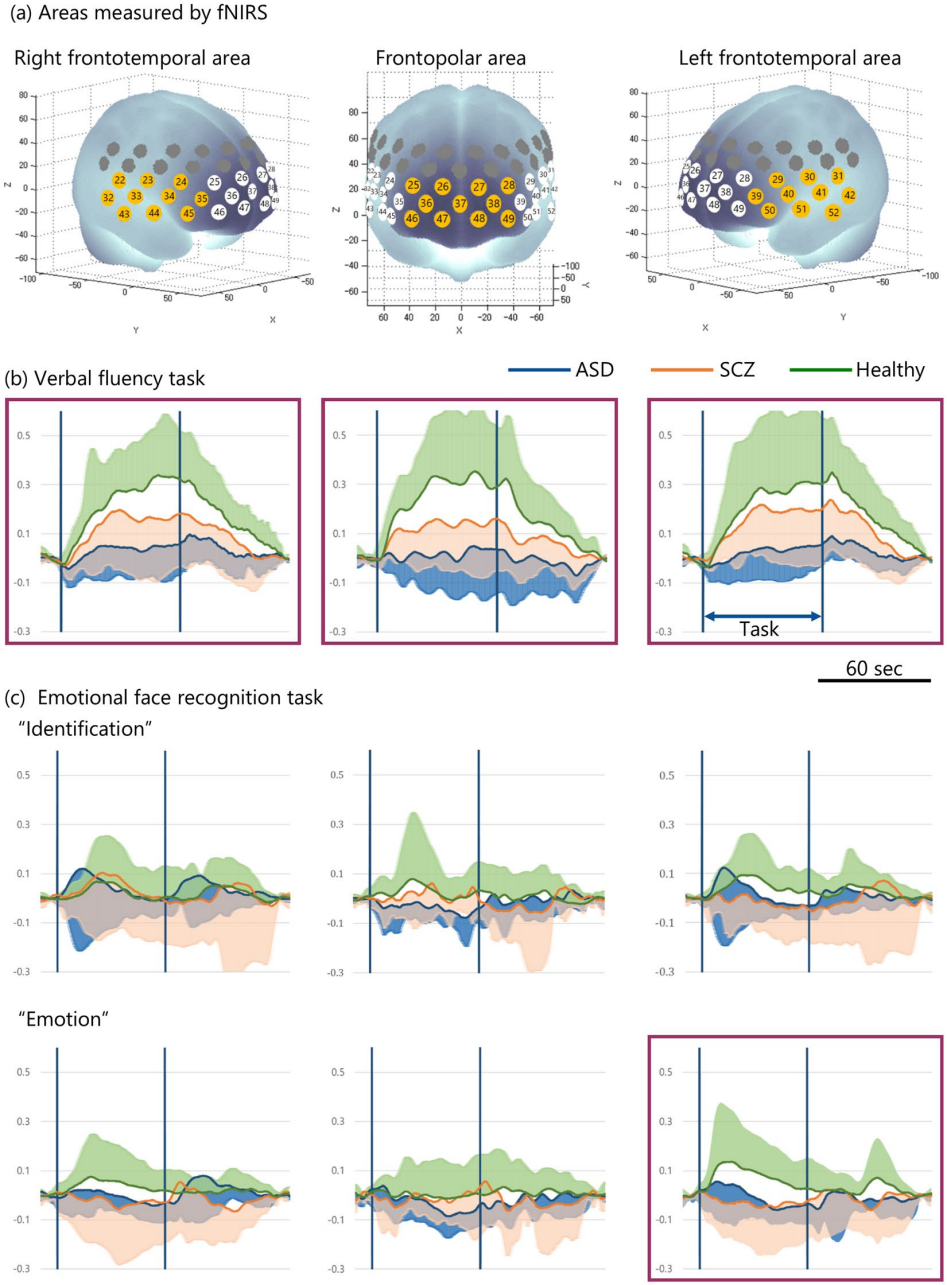
The brain areas evaluated using functional near-infrared spectroscopy (fNIRS) and change in oxygenated haemoglobin [oxy-Hb] levels over time during the verbal fluency and emotional face recognition tasks. (**a**) Anatomical areas of the brain measured using fNIRS. The numbers in tangerine-coloured circles represent the channels of measurement in the anatomical area as identified in the figure. The frontopolar area (channel #25–28, 36–38, and 46–49) corresponding to the superior and middle frontal gyri, and the right (#22–24, 32–35, and 43–45) and left frontotemporal areas (#29–31,39–42, and 50–52) corresponding to the inferior and middle frontal gyri and the anterior portion of the superior and middle temporal gyri. The mean and standard deviation of [oxy-Hb] in the three areas over time, during the verbal fluency task (**b**) and the ‘identification’ (**c**) and ‘emotion’ tasks in the emotional facial recognition task evaluation. Traces outlined in purple represent data with significant differences in the integral value of [oxy-Hb] during tasks across autistic spectrum disorder (ASD) patients, schizophrenia (SCZ) patients, and healthy subjects using the Kruskal-Wallis test. The shaded colour represents standard deviation.

Figure 1(b and c) illustrates the change in the mean and standard deviation values of the integral value of [oxy-Hb] during the task period in patients with ASD, patients with SCZ, and in healthy subjects. During the verbal fluency task as a non-social cognition, the integral value of [oxy-Hb] showed a significant difference and large effect size across the three groups in the frontopolar (*chi-squared* = 18.3, *p* < 0.01, *η*^2^ = 0.36), the left frontotemporal (*chi-squared* = 9.1, *p* = 0.01, *η*^2^ = 0.20), and the right frontotemporal areas (*chi-squared* = 13.6, *p* < 0.01, *η*^2^ = 0.30) (Fig. 1b). In the post-hoc analysis, patients with ASD showed significantly lower integral value of [oxy-Hb] with large effect size compared to healthy subjects in the three areas, and compared to patients with SCZ in the frontopolar area (Table 3). During the emotional facial recognition task as a social cognition, the integral value of [oxy-Hb] showed no significant difference and small to medium effect size during the ‘identification’ task across the three groups in any area, but a significant difference and large effect size during the ‘emotion’ task across the three groups in the left frontotemporal area (*chi-squared* = 6.40, *p* = 0.04, *η*^2^ = 0.14) (Fig. 1c). Patients with ASD showed significantly lower integral value of [oxy-Hb] with large effect size compared to healthy subjects in the left frontotemporal area in the post-hoc analysis (Table 3).

**Table 3:** Post-hoc analysis for the fNIRS results.

	Left frontotemporal	Frontopolar	Right frontotemporal
**Verbal fluency task**			
ASD vs. Healthy	***chi-squared*** = **7.86**, ***p*** < **0.01**, ***η***^**2**^ = **0.26**	***chi-squared*** = **14.5**, ***p*** < **0.01**, ***η***^**2**^ = **0.48**	***chi-squared*** = **12.7**, ***p*** < **0.01**, ***η***^**2**^ = **0.42**
SCZ vs. Healthy	*chi-squared* = 0.63, *p* = 0.43, *η*^2^ = 0.02	***chi-squared*** = **4.70**, ***p*** = **0.03**, ***η***^***2***^ = **0.15**	*chi-squared* = 3.81, *p* = 0.05, *η*^*2*^ = 0.12
ASD vs. SCZ	*chi-squared* = 5.41, *p* = 0.20, *η*^*2*^ = 0.20	***chi-squared*** = **5.20**, ***p*** = **0.02**, ***η***^***2***^ = **0.19**	*chi-squared* = 3.66, *p* = 0.06, *η*^*2*^ = 0.14
**Facial emotional recognition task**			
**Emotion**			
ASD vs. Healthy	***chi-squared*** = **5.77**, ***p*** = **0.02**, ***η***^***2***^ = **0.19**	—	—
SCZ vs. Healthy	*chi-squared* = 3.40, *p* = 0.07, *η*^*2*^ = 0.11	—	—
ASD vs. SCZ	*chi-squared* = 0.06, *p* = 0.80, *η*^*2*^ = 0.02	—	—

Kruskal Wallis test results. ASD, Autistic spectrum disorder; SCZ, schizophrenia. Statistically significant results appear in bold.

### Cognitive brain activation and clinical association

**Figure 2:**
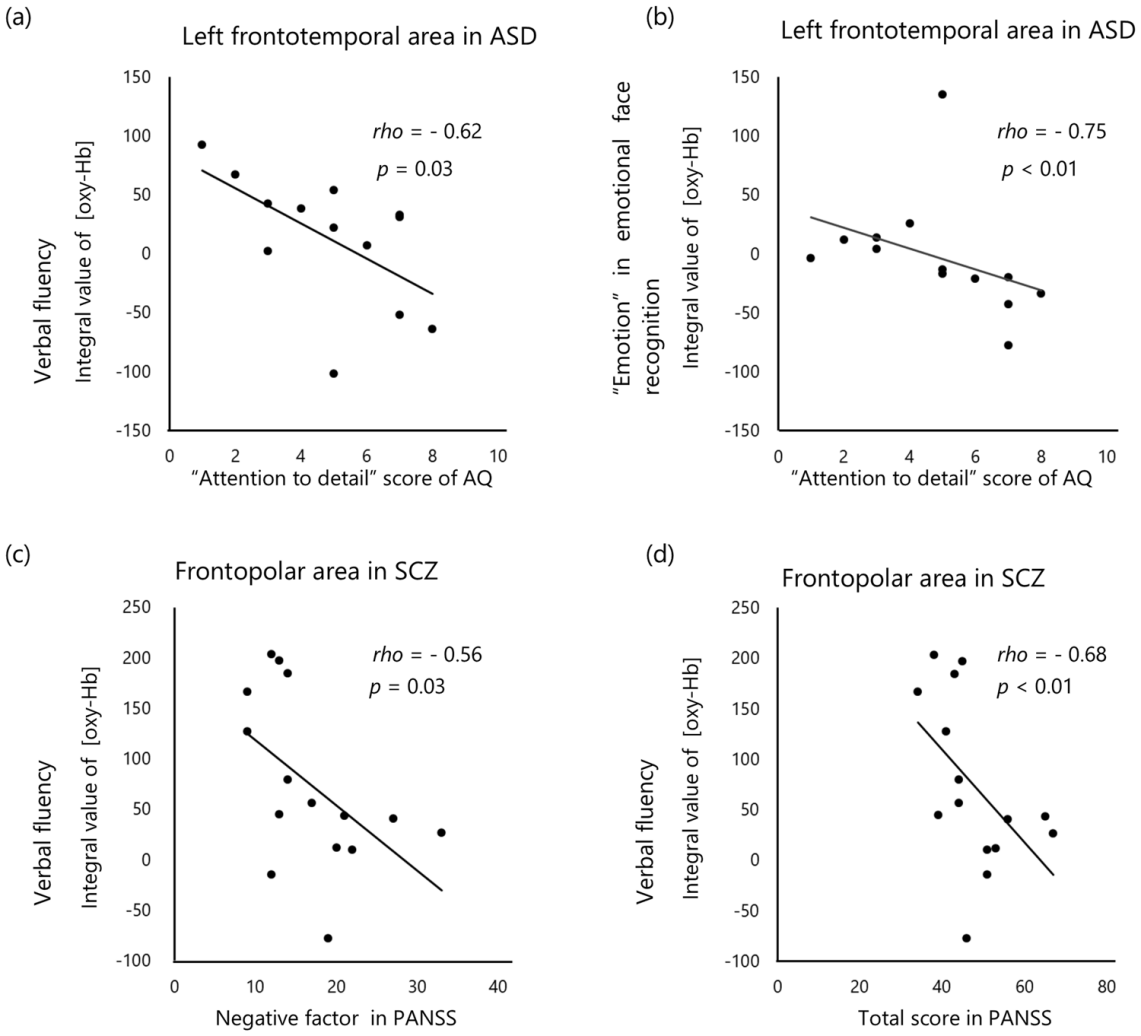
Correlation analysis and scatter plots, and regression analysis of clinical variables and the change in integral value of oxygenated haemoglobin [oxy-Hb]. (**a**) The score in ‘attention to detail’ in the Autism Spectrum Quotient (AQ) and the verbal fluency test, and (**b**) the result of the ‘emotion’ task of emotional face recognition task in patients with autistic spectrum disorder (ASD). The score pertaining to negative factors (**c**) and total (**d**) score as per the Positive and Negative Syndrome Scale (PANSS) and the verbal fluency test in patients with schizophrenia (SCZ). The value of r represented the correlation coefficient by Spearman’s rho method.

We also examined the association of the NIRS results and clinical variables with correlation analysis in the same manner used for the behavioural results and clinical variables. The integral value of [oxy-Hb] in the left frontotemporal area during the verbal fluency task and ‘emotion’ in the emotional face recognition task was significantly and inversely linked to the score for ‘attention to detail’ in AQ in patients with ASD (*rho* = −0.62, *p* = 0.03; *rho* = −0.75, *p* < 0.01, respectively) (Fig. 2a and b). The latter result remained significant after Bonferroni correction. There was no significant correlation between the integral value of [oxy-Hb] in this area during any task and the scores of any other AQ subscale. In patients with SCZ, the integral value of [oxy-Hb] in the frontopolar area correlated significantly and inversely with the negative symptoms score and total score on the PANSS (*rho* = −0.56, *p* = 0.03; *rho* = −0.68, *p* < 0.01, respectively) (Fig. 2c and d). The latter result remained significant after Bonferroni correction. There was no significant correlation between the integral value of [oxy-Hb] during any task in any area and medication load or the other clinical variables in patients with ASD or SCZ.

Exploratory discrimination analyses using the integral value of [oxy-Hb] in the regions with statistically significant differences revealed that ASD (present) vs. SCZ displayed: correct classifications: 75.0%, sensitivity: 84.6%, specificity: 66.7%, LR+: 2.54 of, LR−: 0.23, and DOR: 11.0; ASD (present) vs. healthy showed: correct classifications: 83.9%, sensitivity: 92.3%, specificity: 77.8%, LR+: 4.15, LR−: 0.10, and DOR: 42.0; SCZ (present) vs. healthy revealed: correct classifications: 54.5%, sensitivity: 66.7%, specificity: 55.6%, LR+: 1.50, LR−: 0.60, DOR: 2.50.

## Discussion

Our primary results included: 1) left frontotemporal dysfunction during the social and non-social cognitive tasks and frontopolar and right frontotemporal dysfunction during the non-social cognitive task were found in patients with ASD compared to healthy subjects, 2) frontopolar dysfunction during the non-social task was found in patients with SCZ compared to healthy subjects, and 3) frontopolar dysfunction during the non-social cognitive task differed between patients with ASD and patients with SCZ. The first observation can highly discriminate between the two conditions (ASD versus healthy). Further, the patients with ASD with exaggerated ‘attention to detail’ scores pertaining to the AQ had a relatively poor brain response in the left frontotemporal area during the social cognitive task, and patients with SCZ with more severe symptoms (higher total PANSS scores) had lower brain activation during the non-social cognitive task in the frontotemporal area. These results suggest that the left frontotemporal and frontopolar areas are involved in the distinctive pathophysiology of social and non-social cognitive processing in ASD and SCZ.

Literature evidence suggests that ASD is associated with dysfunction of the left frontotemporal area. An fMRI study revealed that patients with ASD showed reduced left inferior frontal activation during an emotional facial recognition task compared to healthy subjects^25^. Carter *et al*., using fMRI, showed that children with ASD had reduced brain activation in the left inferior frontal gyrus and the superior temporal pole, as well as in the bilateral posterior superior temporal sulcus, insula, and other medial frontal and temporal regions, in response to a social judgment task, compared to typically developed children^26^. Suda *et al*., in an fNIRS study, revealed that healthy participants with higher AQ scores showed less activation in the left superior temporal gyrus during a face-to-face conversation task, which evaluated real-world-like social cognition^27^. These investigators propose that reduced brain activation in the left inferior frontal and superior temporal areas may be due to reduced language processing in a social context in patients with ASD^26^ and in healthy subjects with autistic traits^27^. These findings partly support our results, which showed that patients with ASD had reduced brain activation in response to non-social and social cognition in the left brain areas corresponding to the inferior and middle frontal gyri and anterior superior and middle temporal gyri. Together with the findings of previous studies, the findings in the present study suggest that this area is associated with blunted social cognition skills and language processing in ASD.

This study also found that exaggerated ‘attention to detail’ was associated with poorer brain activation in the left frontotemporal area in response to social cognitive function in patients with ASD. The inferior prefrontal gyrus (both left and right sides) is part of the temporoparietal junction-ventral frontal cortex network involved in stimulus-driven control^28^, called the ‘salience/ventral attention network’^29^. This area plays a crucial role in integrating information from both the external environment and from within the body and in collecting and processing this information^30^. The area is also known to be a key player in cognitive and affective theory of mind^31,32^. A meta-analysis of task-evoked functional MRI studies^33^ with patients with ASD suggested that there is hypoactivation of the frontotemporal area, including the inferior prefrontal gyrus, in response to social cognition and a large-scale, brain network resting state functional MRI study^34^ with the same patient population found that there was hyper-functional connectivity in the salience, frontotemporal, and other networks. Based on such evidence, it can be inferred that possible reduced brain activation of the left frontotemporal area may be associated with abnormal function of attention in patients with ASD. However, as we did not examine other brain areas in the attention network in this study, future studies to address the issue will be required to evaluate brain activation during a variety of attentional tasks using neuroimaging modalities such as fMRI.

The current study found that low brain activation in the frontopolar area was observed in patients with SCZ during a verbal fluency task, and was associated with severity of disease. This finding is consistent with the findings of prior fNIRS studies^19,35,36^. This abnormal frontopolar response was different between patients with SCZ and those with ASD, suggesting that frontopolar dysfunction during this task is potentially a distinctive feature of SCZ and can be used to differentiate between the brain pathophysiology of the two disorders.

Some limitations of this study should be noted. First, the small sample size resulted in limited statistical power, although non-parametric statistical analyses were applied. Second, the study did not evaluate other brain regions, such as the fusiform gyrus and the amygdala (crucial for facial recognition)^37^, due to the shortcoming that fNIRS only measures the hemodynamic response at the surface of the brain. Thus, our results did not reveal whether the activation of other brain areas would influence the differential brain activation pattern observed in patients with ASD and SCZ. Third, as fNIRS has a low spatial resolution (approximately 3 cm, which is roughly 1 gyrus of the brain)^27^, anatomical identification is not as accurate as that obtained in fMRI investigations. Thus, we were only able to delineate the three broad areas for statistical analysis in this study. Fourth, patients with ASD and SCZ were being treated with diverse dosages and types of psychotropic medications during the study, which may have affected the results. However, a meta-analysis study showed that there was no significant difference in behavioural performance of facial emotion perception between medicated and unmedicated patients with SCZ^38^. In addition, our results showed that medication load was not significantly linked with behavioural performance or brain activation during the tasks in patients with ASD or SCZ. Fifth, psychiatric comorbidities may have influenced the results in patients with ASD. Then, we preliminary tested the differences in the behavioural and fNIRS results across patients with ASD with psychiatric comorbidities (n = 8), patients with ASD without psychiatric comorbidities (n = 5), patients with SCZ, and healthy subjects (the results are presented in the Supplementary Information). The result of the fNIRS study during the verbal fluency and emotional facial recognition tasks when comparing patients with ASD without psychiatric comorbidities and healthy subjects did support the results of the original comparison (all patients with ASD versus healthy subjects). These results suggest that the fNIRS data of patients with ASD with psychiatric comorbidity may not have contaminated the results of the fNIRS study procured in the original comparison. However, numerous results were procured with pairwise comparisons (patients with ASD without psychiatric comorbidities versus patients with SCZ, patients with ASD with psychiatric comorbidities versus patients with SCZ, and patients with ASD without psychiatric comorbidities versus patients with ASD with psychiatric comorbidities) (presented in the Supplementary Information). Combined with prior fNIRS studies with patients with mood and anxiety disorders^39,40^, psychiatric comorbidities may not be completely dismissed as potentially influencing the fNIRS or behavioural results. Sixth, we did not use a structured diagnostic interview such as the Autism Diagnostic Interview-Revised or Autism Diagnostic Observation Schedule for patients or healthy subjects. Although we believed that the senior psychiatrists were competent in diagnosing ASD, the use of structured interviews would had additionally allowed for the diagnoses to be validated. Seventh, it would be difficult to generalise the results for patients with ASD and SCZ since approximately half of the patients with ASD had mildly severe ASD characteristics, and may have been relatively high-functioning, while the patients with SCZ were chronically schizophrenic. In addition, the current IQ in patients with SCZ may have affected the results because neurocognitive deficits are associated with social cognition and functional outcome^41,42^. However, we did not assess current IQ. Last, we did not analyse behavioural performance and brain activation with reference to individual emotions like sadness, anger, and fear in the emotional face recognition task, due to the small sample size, which was not conducive to sufficiently powered statistical analyses. Future studies investigating detailed brain activation responses to each emotional stimulus using a larger sample size are indicated. Eighth, we did not use the same clinical assessment for patients with ASD and patients with SCZ, e.g. the Brief Psychiatric Rating Scale. Moreover, the AQ is a self-rated questionnaire, which could be prone to subjective bias, while the PANSS is a clinician-rated measure. Therefore, the results of the correlation analysis between AQ and PANSS should be interpreted with caution. Last, we corrected for motion artefacts using the moving average^35^ and algorithm methods^19^ and by deleting channels with remarkable artefacts, as assessed by an expert. However, as algorithms to correct for motion artefacts in fNIRS data have been continuously advancing^43,44^, it would be necessary to use another algorithm to more accurately calibrate for artefacts in the NIRS signals in future fNIRS studies.

The current study demonstrated that patients with ASD showed reduced brain activation of the left frontotemporal area for non-social and social cognition, which was associated with ‘exaggerated attention’. Patients with SCZ showed brain dysfunction in the frontopolar area for non-social cognition, which was associated with severity of symptoms. These findings indicate that the two areas of the brain are involved in the differential brain pathophysiology relevant to cognitive processing in ASD and SCZ.

## Methods

### Participants

We studied 26 patients (13 with ASD and 13 with SCZ) and 18 healthy subjects (Table 1). Premorbid IQ was evaluated with the Japanese version of the National Adult Reading Test^45^. All participants were right handed^46^. Patients with ASD and SCZ were recruited at the Yamaguchi University Hospital, Yamaguchi Prefectural Mental Health Medical Center, and the Katakura Hospital. Healthy subjects were recruited by advertisements and word-of-mouth communication in the community. Several patients with SCZ and several healthy subjects who participated in this study had also participated in our prior studies^22,23^. This study was approved by the Institutional Review Boards of the three sites and all experiments were performed in accordance with relevant guidelines and regulations. After the study was fully described to each subject, we obtained written informed consent from all participants. The patients met the Diagnostic and Statistical Manual of Mental Disorders, 5th edition (DSM-5)^1^ criteria for ASD or SCZ, as assessed by senior psychiatrists during clinical interviews. They were also screened with the Mini-International Neuropsychiatric Interview (M.I.N.I.)^47^ to evaluate psychiatric comorbidities. Five patients with ASD and one patient with SCZ had psychiatric comorbidities: for ASD, two had social anxiety disorder, two had major depressive disorder, one had dysthymia, panic disorder, social anxiety disorder, general anxiety disorder, and obsessive-compulsive disorder; for SCZ, one had obsessive-compulsive disorder. Any participant who had a neurological illness, a history of traumatic brain injury with loss of consciousness, history of alcohol or drug abuse, or any physical illness such as hepatitis, brain tumour, or epilepsy, was excluded from the study. Healthy subjects were screened using the M.I.N.I. We excluded healthy participants with first- or second-degree relative(s) with a history of psychiatric disorders. All included patients were receiving psychiatric medications during the study, except for two unmedicated patients with ASD. Symptomatic assessments were evaluated as per the Autism Spectrum Quotient (AQ)^48^ for patients with ASD, and the Positive and Negative Syndrome Scale (PANSS) for psychiatric symptoms^49^ for patients with SCZ. Approximately half of the patients with ASD had lower AQ total score than the cut-off point (31) for screening, which likely means that these patients had mildly severe ASD.

### Task procedure

We measured participant performance in a verbal fluency task and an emotional facial recognition task. The verbal fluency task consisted of a 30-s pre-task baseline period, a 60-s word-production period comprising three 20-s blocks, and a 70-s post-task baseline period^35,36^. During the baseline periods, participants were asked to repeatedly vocalize the five Japanese vowels in order. During the word production period, participants were instructed to generate as many words as possible for a given Japanese mora (rhythmic phonetic unit in the Japanese language). Words were recorded on a digital recorder, and repeats as well as those words inflected for tense or number based on an earlier word were excluded when calculating the total number of words as the measure of task performance.

**Figure 3:**
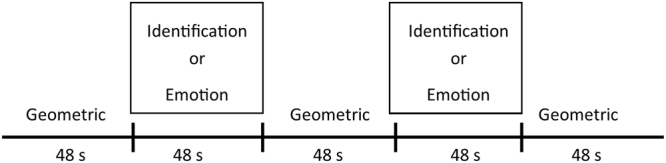
Task design for the emotional face recognition task. The task consisted of an ‘identification task’ (facial sex-matching) and an ‘emotion task’ (facial emotional-matching). The baseline task involved selecting a geometric figure. The presentation of stimuli, choice of target stimuli, and the identification and emotion tasks were administered in a counterbalanced order across subjects.

Details of the emotional facial recognition task have been described in our previous study^22^. The task consisted of an ‘identification task’ to assess face perception, and an ‘emotion task’ to assess facial emotions (Fig. 3). The subject was required to choose which of the two figures in the lower panel matched the figure in the upper panel. In the identification task, sex-matching was required between faces in the upper and lower panels. In the facial emotion task, the participants were asked to match figures in the lower panel with a figure showing similar facial emotions in the upper panel. Sadness, anger, and fear were the facial emotions included in the task because negative emotions minimize heterogeneity due to valence^13^ and show more robust brain activation compared to positive emotions in patients with SCZ^38^ and neurodevelopmental disorders^50^. The baseline task involved selecting the geometric figure in the lower panel that matched that in the upper panel. Figures included were a circle, triangle, and a square. This figure-matching task was used to cancel sensorimotor artefacts due to finger and body movements from the fNIRS signal change during the identification and emotion tasks. Thus, the figure-matching task was eliminated from statistical analyses. There were 12 trials, for a total duration of 300 s. The duration of one block was 60 s for the identification and emotion tasks. The presentation of stimuli, choice of target stimuli, and the identification and emotion task order were administered in a counterbalanced order across subjects. Behavioural performance was assessed by the accuracy and MRT for each task. Participants completed task training in advance of the main task, and were confirmed to have fully learned the task before the study proper commenced.

### NIRS measurements

We applied a continuous-wave fNIRS system (ETG-4000, Hitachi Medical Co., Japan) to measure brain function. Details of the fNIRS system have been described in our previous studies^22,23,51^. Relative changes in the concentrations of oxygenated [oxy-Hb] and deoxygenated haemoglobin were monitored. The time resolution was set at 0.1 s. Multichannel probe holders (3 × 5) were used, each consisting of 17 eliminating and 16 detecting probes alternately arranged at an inter-probe distance of 3 cm, resulting in 52 channels per set. Channels were placed in accordance with the international 10–20 system. The lowest probes were positioned along the Fp1–Fp2 line. We corrected for motion artefacts using the moving average method, according to previous studies^35^, which removes short-term motion artefacts from the analysed data (moving average window: 5 s), and the algorithm method to exclude channels contaminated with rhythmic signals that indicate noise and motion artefacts^19^. Additionally, the channels with remarkable motion artefacts were deleted based on the blinded assessment of an expert in fNIRS studies (K.M.). The data were analysed using the integral mode, in which the pre-task baseline during the control block was determined as the mean [oxy-Hb] during the 10 s just prior to the task period; the post-task baseline during the control block was determined as the mean [oxy-Hb] of the last 10 s in the post-task period, and the data between two baselines were linearly fitted. As was performed in prior fNIRS studies^19,22,23,52^, we measured the frontal and temporal areas via 31 channels (channels #22 to 52). We anatomically identified areas by a virtual registration method with automated anatomical labelling^53^ that enables NIRS channel positions to be registered in the standard brain space^54^. The 31 channels were classified into three areas according to previous fNIRS studies^19,52^ (Fig. 1a): the frontopolar area (channels #25–28, 36–38, and 46–49) (corresponding to the superior and middle frontal gyri), the right frontotemporal area (#22–24, 32–35, and 43–45), and the left frontotemporal area (#29–31,39–42, and 50–52) (corresponding to the inferior and middle frontal gyri and the anterior portion of the superior and middle temporal gyri).

### Statistical analysis

We analysed the three participant groups on behavioural performance in the verbal fluency and the emotional facial recognition tasks using the Kruskal-Wallis Test. Subsequently, we compared the results between each two groups at the post-hoc level and tested the effect size (*η*^2^) of the results. We also tested the correlation between clinical variables and performance parameters with significant differences between patients with ASD and healthy subjects and between patients with SCZ and healthy subjects using Spearman’s rho method.

For analysis of the fNIRS data, [oxy-Hb] during the task was used as an outcome measure for statistical analyses, since it is thought to reflect the activation of grey matter in the brain^55^. The change in [oxy-Hb] over the period of the targeted task, termed the integral value of [oxy-Hb], was used for analysis^19^. We analysed the integral value of [oxy-Hb] in the verbal fluency and emotional facial recognition tasks across the three participant groups using the Kruskal-Wallis test. Subsequently, we analysed the results between each two groups at the post-hoc level and tested the effect size (*η*^2^) of the results. We performed correlation analysis of clinical variables and the integral value of [oxy-Hb] in the areas with significant differences between patients with ASD and healthy subjects and between patients with SCZ and healthy subjects using Spearman’s rho method.

We exploratively performed Fisher’s linear discrimination analyses of the three groups using the integral value of [oxy-Hb] during the verbal fluency and emotional face recognition tasks in the frontopolar, and left and right frontotemporal regions that showed significance by group-comparison analyses. The results are presented using the following parameters: correct classification (%), sensitivity (%), specificity (%), likelihood ratio of a positive test result (LR+), likelihood ratio of a negative test result (LR−), and diagnostic odds ratio (DOR).

The datasets generated and/or analysed during the current study are available from the corresponding author on reasonable request.

## Electronic supplementary material

Supplementary Information
