# Supplementary material for: Differences in frontotemporal dysfunction during social and non-social cognition tasks between patients with autism spectrum disorder and schizophrenia
Source: Sci Rep. 2018 Feb 14;8:3014. doi: 10.1038/s41598-018-21379-w (PMC5813031; doi:10.1038/s41598-018-21379-w)
Supplement: Supplementary file 1 — Supplementary Information [file 41598_2018_21379_MOESM1_ESM.pdf]

Supplementary Information

Differences in frontotemporal dysfunction during social and non-social cognition tasks between patients with autism spectrum disorder and schizophrenia

Keiko Hirata, Kazuteru Egashira, Kenichiro Harada, Mami Nakashima, Masako Hirotsu, Shinji Isomura, Toshio Watanuki, Toshio Matsubara, Yoichi Kaku, Hiroshi Kaneyuki, Yoshifumi Watanabe, Koji Matsuo

We compared behavioural and fNIRS data during the verbal fluency task and emotional facial recognition task across patients with ASD without psychiatric comorbidities, patients with ASD with psychiatric comorbidities, patients with SCZ, and healthy subjects with the Mann-Whitney U test, to evaluate the effect of psychiatric comorbidities in patients with ASD.

To confirm the results of fNIRS in the original comparison (patients with ASD vs. healthy subjects), we compared fNIRS data during the verbal fluency task and emotional facial recognition task between patients with ASD without psychiatric comorbidities and healthy subjects. The new comparison showed significant results in the same area during the verbal fluency task and a larger area during the emotional facial recognition task in relation to the

original comparison; for instance, patients with ASD without psychiatric comorbidities showed significantly smaller integral value of [oxy-Hb] during the verbal fluency task in the three areas (Frontopolar, U = 21.0, p = 0.005; left frontotemporal, U = 35.0, p = 0.04; right frontotemporal, U = 26.0, p = 0.011), and significantly smaller integral value of [oxy-Hb] during the emotional facial recognition task in the left (U = 29.0, p = 0.017) and right frontotemporal areas (U = 34.0, p = 0.035).

Patients with ASD without psychiatric comorbidities showed significantly more generated words in the verbal fluency task (U = 21.5, p = 0.013) compared to patients with SCZ.

Patients with ASD with psychiatric comorbidities showed significantly smaller integral value of [oxy-Hb] during the verbal fluency task in the three areas compared to patients with SCZ (Frontopolar, U = 10.0, p = 0.016; left frontotemporal, U = 10.0, p = 0.016; right frontotemporal, U = 10.0, p = 0.016) and in the right frontotemporal area compared to patients with ASD without psychiatric comorbidities (U = 4.0, p = 0.019). There were no other differences in the behavioural or fNIRS data during the verbal fluency task and emotional face recognition task in the other group comparisons.
